# Water Access and Adherence Intention Among HIV-Positive Pregnant Women and New Mothers Receiving Antiretroviral Therapy in Zambia

**DOI:** 10.3389/fpubh.2022.758447

**Published:** 2022-04-01

**Authors:** Jerry John Nutor, Shannon Marquez, Jaime C. Slaughter-Acey, Thomas J. Hoffmann, Rose Ann DiMaria-Ghalili, Florence Momplaisir, Emmanuel Opong, Loretta Sweet Jemmott

**Affiliations:** ^1^Department of Family Health Care Nursing, School of Nursing, University of California, San Francisco, San Francisco, CA, United States; ^2^Undergraduate Global Engagement, Columbia University, New York City, NY, United States; ^3^Division of Epidemiology and Community Health, University of Minnesota, Minneapolis, MN, United States; ^4^Department of Epidemiology and Biostatistics, and Office of Research, School of Nursing, University of California, San Francisco, San Francisco, CA, United States; ^5^College of Nursing and Health Professions Drexel University, Philadelphia, PA, United States; ^6^School of Medicine, University of Pennsylvania, Philadelphia, PA, United States; ^7^World Vision Swaziland, Mbabane, Eswatini

**Keywords:** ARV, Sub-Saharan Africa, potable water, women, rural, borehole, well water, Theory of Planned Behavior

## Abstract

**Background:**

Mother-to-infant transmission of HIV is a major problem in Sub-Saharan Africa despite free or subsidized antiretroviral treatment (ART), but is significantly reduced when mothers adhere to ART. Because potable water access is limited in low-resource countries, we investigated water access and ART adherence intention among HIV-positive pregnant women and new mothers in Zambia.

**Methods:**

Our convenience sample consisted of 150 pregnant or postpartum women receiving ART. Descriptive statistics compared type of water access by low and high levels of ART adherence intention.

**Results:**

Most (71%) had access to piped water, but 36% of the low-adherence intention group obtained water from a well, borehole, lake or stream, compared to only 22% of the high-adherence intention group. The low-adherence intention group was more rural (62%) than urban (38%) women but not statistically significant [unadjusted Prevalence Ratio (PR) 0.73, 95% CI: 0.52–1.02; adjusted PR 1.06, 95% CI: 0.78–1.45].

**Conclusion:**

Providing potable water may improve ART adherence. Assessing available water sources in both rural and urban locations is critical when educating women initiating ART.

## Introduction

Globally, women represent more than half the number of people living with human immunodeficiency virus (HIV), and the majority of cases are in developing countries within Sub-Saharan Africa, including Zambia. For example, in 2019 it was reported that Sub-Saharan Africa had 52.6% (20 million) of all HIV-positive cases in the world, and over 59% of these cases were women (1). A majority of these women are in their reproductive years, which has led to high rates of vertical transmission of HIV from mother to baby. Vertical transmission of HIV can occur during the process of delivery, through breastfeeding, or even during the pregnancy itself. Vertical transmission without medical intervention ranges between 15 and 45%, depending on risk factors such as high viral load, low CD4 count, mixed infant feedings, anemia, and hospitalization after childbirth (2, 3). However, with appropriate and timely medical intervention, the rate of infant infections can be substantially reduced (3).

In an effort to eliminate vertical transmission of HIV, the World Health Organization (WHO) recommends a broad approach that includes: a) preventing HIV infection among women of reproductive age, b) preventing unintended pregnancies among women living with HIV, and c) providing appropriate treatment, care and support to mothers living with HIV and their children and families (4, 5). As of 2019, over 95% of HIV-positive pregnant women in Eastern and Southern Africa were receiving antiretroviral therapy (ART) (1). In Zambia, pregnant women are routinely tested for HIV during prenatal visits. Beginning in 2012, pregnant women testing positive for HIV are started on a life-long fixed combination of ART at the time of diagnosis (6). In 2018 Zambia also adopted the latest WHO recommendation for a fixed-dose combination of Dolutegravir 50 mg, Tenofovir 300 mg, and Lamivudine 300 mg as the preferred first-line ART for HIV infection in all patients irrespective of CD4+ or viral load (7, 8).

In two separate cross-sectional studies, HIV-positive pregnant and lactating mothers in Zambia were found to have between 75 and 82% adherence to ART (9, 10). These and other studies have also indicated an increased uptake of ART among pregnant and lactating women in Zambia (11, 12). Although the increase in enrollment of pregnant women on ART has come as good news for eliminating vertical transmission of HIV and improving overall health of HIV-positive mothers, there is concern about inadequate access to resources such as potable water, adequate sanitation and healthy nutrition that may influence women's ability to adhere to ART (13). For example, it is estimated that only 40–60% of people in low-resource countries, including Sub-Saharan Africa, have access to clean water (14). As of 2018, only 64% of Zambia's population had access to potable water, with urban areas having more access (87%) than rural areas (49%) (15). The government of Zambia and a few not-for-profit organizations such as United Nations International Children's Emergency Fund (UNICEF) have been working to increase access to potable water in rural and peri-urban communities throughout Zambia (16).

Demand for water increases among pregnant and breastfeeding women regardless of their HIV status (17), however, those who are HIV positive have a significantly higher demand for household water (18). The higher demand for water may result from instructions to women on the importance of adequate hydration while taking ART, but is also related to managing unpleasant side effects associated with ART, such as diarrhea, nausea and vomiting (19–21). Without adequate access to water for domestic use, women experiencing these side effects could skip their medication to avoid the struggle to find water. In one cross-sectional study conducted in Zambia, HIV-positive pregnant women and lactating mothers with access to a toilet in the home were more likely to adhere to ART compared to women without a toilet facility in the home (21). In another study, 62% of pregnant women enrolled in an ART program in Zimbabwe were not adherent to their ART regimen at 1year (22). Poor adherence to ART may lead to reduced drug efficacy and increased risk of vertical transmission of HIV at the individual level (23), and may lead to the development of drug-resistant mutations at the population level (19, 21).

Types of adherence to medication can be categorized as intentional and unintentional. Intentional poor adherence occurs when a patient decides not to take the medication as prescribed usually because of negative attitudes and beliefs and lack of knowledge about transmission of the disease. Unintentional poor adherence, however, may occur when a patient intends to take the medication, but is prevented from taking it due to challenges in relation to capacity and resources such as forgetfulness, poor comprehension, cost of medication, or no access to food or water (21, 24–26). Unintentional poor adherence has been increasing in low resource countries in the past few years (24) for reasons unknown. To address the factors influencing ART adherence in low resource countries, there is a need to better understand intention to adhere. Adherence to ART among pregnant women and new mothers is a function of their intention to take the antiretroviral drugs and their knowledge about HIV transmission to others, but can also be associated with access to potable water needed for cooking, flushing toilets, and maintaining personal hygiene when they experience common side effects of ART (18, 21). People in rural areas are reported to have low adherence to ART (24), and in Zambia, rural areas generally have less access to water, which may affect adherence. However, the association between adherence intention and water access has not been previously examined (15).

Intent to take medication is influenced by various factors, with water being one of the most critical factors. Women receiving ART may intend to take the medication when they evaluate that behavior as positive, believe there is enough water for them to take care of themselves, and feel confident they can perform the behavior without difficulty. Therefore, we sought to understand the relationship between water access and adherence intention among HIV-positive pregnant women and new mothers receiving ART in Zambia, a country with one of the highest prevalence rates of HIV, and where over 80% of all pregnant women are currently on ART (27, 28).

## Materials and Methods

### Design and Sample

This is a multi-center cross sectional study of 150 HIV-positive pregnant and new mothers receiving ART in urban (Lusaka) and rural (Sinazongwe) districts of Zambia. Detailed descriptions of the sample are published elsewhere (9, 21, 23). We recruited women who sought antenatal and postnatal care in government hospitals/health centers in these districts using a convenience sampling method. The sites for the current study include three hospitals in the urban area, and one hospital and two health centers in the rural area. These hospitals and health centers are the main designated ART clinics for antenatal and postnatal care for pregnant women and new mothers living with HIV in the selected districts. Women were eligible to participate in the study if they were at least 18 years of age, had no intellectual impairment noted in their medical record), pregnant or breastfeeding, and had initiated ART. Women were excluded from the study if they were critically ill (defined as a recent hospitalization) or enrolled in ART for <2 months.

### Recruitment and Data Collection

Data for the study were collected between June and August 2017. Pregnant women and new mothers living with HIV were contacted in person in the consulting room when they arrived for a clinic visit to collect their ART drugs or to see their healthcare provider for an appointment. The healthcare provider talked to potential participants about the current study. Potential participants who agreed to learn more about the study were then approached by the trained research assistant who explained the purpose, benefits and risks, and confidentiality associated with participation. Women who could read were given a copy of the information sheet to help them understand details of the study. Women who could not read had details on the information sheet explained by the research assistant in a language (Tonga or Nyanja) that they understood. After addressing any concerns or questions from potential participants, they were asked to make a voluntary decision on whether to participate. Women who agreed to participate signed an informed consent form. The acceptance rate was 100%; all eligible women approached by the research assistants agreed to participate in the study.

Data collection by the trained research assistants took place in either a consulting room or a private room in the hospital/health center. Participants who could read and write were given the self-administered questionnaire and participants who could not read or write had face-to-face interviews to obtain responses on the questionnaire. The average time for completing the questionnaire was 20–35 min.

### Measures

#### ART Adherence Intention

The outcome variable, ART adherence intention, was measured using four items on a 5-point Likert scale (ranging from 1 = strongly disagree to 5 = strongly agree). Based on the Theory of Planned Behavior, general items are suggested that can be adapted for specific clinical situations (29, 30). The items underwent revision and pilot testing with 10 pregnant women and new mothers living with HIV. Findings from the pilot test were used to finalize the items. The four behavioral adherence items were: *(i) I plan to take my medication consistently as prescribed by the healthcare provider, (ii) I intend to take my medication consistently as prescribed by the healthcare provider, (iii) I want to take my medication consistently as prescribed by the healthcare provider, (iv) I will take my medication consistently as prescribed by the healthcare provider*. Responses to the four items created an adherence intention score that ranged from 4 (low intention) to 20 (high intention). Based on the summary score median split, participants were categorized into two groups: 1) low adherence intention with a score < median 12, or 2) higher adherence intention with a score ≥ median. The four items from this instrument had a reliability coefficient (α) of 0.80, indicating acceptable internal consistency.

#### Independent Variables

The independent variable was type of water access. Water access was categorized according to the source of household water. Women were asked to report the type of water source available (1 = piped water in the home or community, or 2 = borehole, well or surface water including stream, lake or dam). Because water access varies greatly by urban and rural locality (14, 31), women were also compared by household residence located in an “urban” or “rural” district.

#### Covariates

The participant's knowledge of HIV transmission was assessed using a thirteen-item scale adopted from Ebuy et al. (24). Women responded “true” or “false” on items such as “adhering to antiretroviral drugs can reduce the risk of opportunistic infections.” The HIV transmission knowledge score ranged from 0 to 13, with higher scores representing higher knowledge levels of HIV transmission. Women were categorized into two groups using the 75th percentile: scores below the 75th percentile indicated inadequate or less knowledge about HIV transmission, and scores equal or above the 75th percentile indicated sufficient or high knowledge about HIV transmission. The instrument had acceptable reliability in this sample (Cronbach α = 0.75).

Based on the literature and previous findings, other covariates were also considered. These potential confounders included age, type of employment (employed, housewife, or other such as daily laborer), and monthly household income below 3,000 Zambian Kwacha (equivalent to USD 200) or 3,000 Zambian Kwacha and above.

### Data Analysis

Data were analyzed using STATA version 16.1 (College Station, TX). Descriptive statistics, including frequencies and percentages, means, and standard deviations (SD), were used to summarize the distribution of the adherence intention scores by the independent variable (water source) and covariates (knowledge of HIV transmission, age, type of employment and monthly household income). We wanted to estimate the prevalence ratio of dichotomized adherence intention (score ≥ 17) using a log-linear model, so we fit mixed effects Poisson regression models [with a robust variance estimate as suggested (32)], including a random effect for center/hospital. We first tested covariates individually (including the random effect for center/hospital), and then fit a series of models using a stepwise theoretical approach: Model 1 included only the primary predictor of interest, water access. Model 2 controlled for HIV transmission knowledge, a known predictor of ART adherence. Model 3 adjusted for demographic factors that included age group, occupation, and income in addition to knowledge. Finally, place of residence was added in Model 4 to account for its influence on water access and other potential predictors of adherence intention.

### Ethical Approval and Consent

The study was conducted in accordance with the Declaration of Helsinki for research involving human subjects. Written informed consent was obtained from the women before inclusion in the study and confidentiality was maintained. The protocol for this study was approved by Drexel University and two local institutional review boards: the Eres Converge Institutional Review Board and the Zambia National Health Research Authority.

## Results

### Sample Characteristics and Bivariate Analysis

Table 1 presents characteristics of the total sample. The sample included 150 women with representation from both urban (*n* = 81; 54%) and rural (*n* = 69; 46%) districts. Overall, 71% of the sample had access to piped water source regardless of urban or rural setting. The most common category of access to water was from a community pipe (48%) followed equally by either pipe into the home (33%) or borehole-type access (29%). The overall sample was relatively young (M = 29 ± 6.2 SD years old), with only 39% over 30 years of age. The participants were socio-economically diverse: 85% were below the median monthly income in Zambia (<3,000 Zambian Kwacha or US $200), and 46% described themselves as a housewife not working for pay outside the home.

**Table 1:** Sample characteristics by adherence intention group (*N* = 149).

**Characteristic**	**Total sample**	**Low adherence**	**High adherence**	**Prevalence ratio^a^**	***P*-value**
	**(*N* = 150)**	**intention (*n* = 73)**	**intention (*n* = 77)**	**Estimate (95% CI)**	
	***n* (%)**	***n* (%)**	***n* (%)**	
**Water source**					
Piped water	106 (71)	47 (64)	59 (78)	Ref	
Borehole, other surface water	43 (29)	26 (36)	17 (22)	0.73 (0.52–1.02)	0.066
**HIV transmission knowledge**					
Low (<12)	112 (75)	64 (88)	48 (62)	0.58 (0.47–0.73)	<0.001
High (12–13)	38 (25)	9 (12)	29 (38)	Ref	
**Age (years)**					
18–30	92 (61)	44 (60)	48 (62)	Ref	
Above 30	58 (39)	29 (40)	29 (38)	0.99 (0.61–1.62)	0.98
**Occupation**					
Employed	41 (27)	16 (22)	25 (32)	Ref	
Housewife	69 (46)	36 (49)	33 (43)	0.82 (0.65, 1.03)	0.095
Unskilled labor	40 (27)	21 (29)	19 (25)	0.81 (0.42, 1.60)	0.54
**Household monthly income**					
<3,000 Zambian Kwacha	125 (85)	66 (90)	59 (79)	Ref	
≥3,000 Zambian Kwacha	23 (15)	7 (10)	16 (21)	1.54 (1.38, 1.72)	<0.001
Missing	2				
**Place of residence**					
Urban	81 (54)	28 (38)	53 (69)		
Rural	69 (46)	45 (62)	24 (31)	0.47 (0.09–2.60)	0.39

a *From mixed effects poisson regression with robust variance estimate (i.e., log-linear model), including a random effect for clinic*.

**Figure 1:**
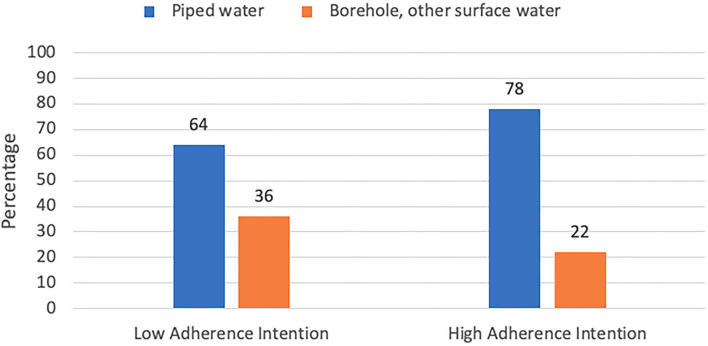
Water access source by adherence intention group.

Table 1 also presents sample characteristics by low and high adherence intention groups. In the low-adherence intention group, 36% had access to a borehole type of water supply and only 22% of the high-adherence intention group accessed a borehole (Figure 1). Access to piped water was predominant for both the low adherence (64%) and high adherence (78%) intention groups. However, more women in the low adherence intention group accessed water from a borehole or other source (36%) compared to the high adherence intention group (22%). This difference did not reach statistical significance (PR 0.73; 95% CI: 0.52–1.02; *p* = 0.066) in the bivariate analysis.

Bivariate analyses did show a significant association between adherence intention and HIV transmission knowledge (PR 0.58; 95% CI: 0.47 – 0.73; *p* <0.001), with only 12% of women in the low adherence intention group scoring high on HIV transmission knowledge compared to 38% of the high intention group scoring high on transmission knowledge. Finally, as seen in Table 1, women in the low ART adherence intention group did not differ on age, occupation, or rural location, but were more likely to have less household income (PR 1.54; 95%CI: 1.38–1.72; *p* < 0.001) compared to the high ART adherence intention group.

### Predictors of Adherence Intention

Given the high potential for confounding relationships between all these demographic factors, multivariable analysis was done to examine the relative effects on high and low adherence intention. A series of multivariable models were fit using a stepwise theoretical approach including additional potential confounders at each step (see Methods). Table 2 presents the results from the Poisson regression analyses of predictors of adherence intention among Zambian women. Water access source was not associated with ART adherence intention in neither unadjusted nor adjusted models that controlled for income and rural or urban residence. When controlling for water source and the other five predictors in the model, women who resided in the rural District of Sinazongwe remained significantly less likely to have adherence intention scores compared to women in the urban District of Lusaka. Income, which was significantly related to adherence intention in bivariate analysis was no longer significant when other variables were included in the multivariable analyses (PR 1.18, 95% CI.78 – 1.78).

**Table 2:** Regression models for predictors of ART adherence intention (*N* = 148).

**Characteristics**	**Model 1**	**Model 2**	**Model 3**	**Model 4**
	**PR (95% CI)^a^**	**PR (95% CI)^a^**	**PR (95% CI)^a^**	**PR (95% CI)^a^**
**Water source**				
Piped water	Referent	Referent	Referent	Referent
Borehole or surface water	0.73 (0.52–1.02)	0.77 (0.52–1.14)	0.80 (0.61–1.05)	1.06 (0.78, 1.45)
**HIV transmission knowledge**				
High (12–13)		Referent	Referent	Referent
Low (<12)		0.59 (0.49,0.70)^*^	0.62 (0.49–0.79)^*^	0.65 (0.50,0.84)^*^
**Age**				
18–30			Referent	Referent
Above 30			1.02 (0.65–1.60)	1.06 (0.67, 1.68)
**Occupation**				
Employed			Referent	Referent
Housewife			0.96 (0.80–1.15)	0.84 (0.58, 1.23)
Unskilled labor			1.01 (0.49, 2.06)	1.13 (0.61, 2.06)
**Household monthly income**				
≥3,000 Zambian Kwacha			Referent	Referent
<3,000 Zambian Kwacha			1.31 (1.08, 1.61)	1.18 (0.78, 1.78)
**Place of residence**				
Urban				Referent
Rural				0.53 (0.24–1.15)
Pearson's and Deviance goodness of fit tests both had P = 1.00 for all four models.^b^

* *p-value ≤ 0.05.*

a *From mixed effects Poisson regression with robust variance estimate (i.e., log-linear model), including a random effect for clinic. PR, prevalence ratio.*

b *From fixed effects Poisson regression model (with a fixed effect for clinic, since goodness of fit tests are not implemented for random effects models; model coefficients negligibly different)*.

In the multivariable models, HIV transmission knowledge was consistently significant as a predictor of adherence. Compared to women with more knowledge about transmission, women with less knowledge were less likely to be in the high adherence intention group (PR 0.65, 95% CI: 0.50 – 0.84) after controlling for other variables in the final model.

## Discussion

Results from this study provide a better public health understanding of the role that water access plays in ART adherence intention among HIV-positive women in urban and rural districts of Zambia. To our knowledge, this is the first study to investigate this relationship in Zambia. High ART adherence intention differed by residential location, which was an expected finding given the limited access to potable water in rural districts of Zambia where a borehole or water from a lake or stream is more common. Findings from this study suggest that despite adequate knowledge about HIV transmission, limited access to potable water in rural areas place women who are pregnant and breastfeeding women at higher risk of poor ART adherence.

Findings from our study are consistent with reported rates of access to potable water in Zambia. For example, in a national survey it was reported that access to potable water differed by rural-urban residence with only 49% of rural dwellers having access to water compared to 87% of urban dwellers (15). Lack of potable water can negatively affect women living with HIV/AIDS, especially when they are pregnant or breastfeeding. Therefore, a possible explanation for ART adherence intention rates in our study could be the variability in access to potable water according to place of residence in Zambia. Also, the women in our study might experience some of the common side effects of ART and decided not to take their medication due to the lack of easy access to potable water. In addition to diarrhea as a side effect of some ART (19, 33), diarrhea is a common experience for HIV-positive adults with compromised immune systems (33). According to previous studies, up to 90% of people living with HIV in low-resource countries suffer from chronic diarrheal diseases. These findings help to explain the increased demand for water among people living with HIV, especially HIV-positive women who are pregnant or breastfeeding.

The demand for potable water is high among pregnant and breastfeeding women regardless of their HIV status (17, 34). Women who are HIV-positive have a significantly higher need for household water (18), yet even in some low-resource countries where water is readily accessible there are risks of water contamination (14). For example, running water could stop for days or even weeks in homes with piped water, forcing the community to use unsafe water sources for cooking and other domestic purposes (35). Also, even when boreholes or wells are built and water sanitation facilities are developed in rural areas, they may be improperly maintained due to limited financial resources (36). This situation makes it difficult for pregnant and lactating women to access adequate water, resulting in avoidance or skipping a medication dosage, especially if diarrhea-related side effects are also experienced (26). For these women to have high adherence intention to ART, basic needs must first be met, including access to a safe potable source of water.

An important finding in this study is the strong association between adherence intention and place of residence, even with controlling for confounding issues of source of potable water, income and knowledge about the transmission of HIV infection. Results in our study indicated that it cannot be assumed that women who live in urban areas have better access to potable water piped into the home compared to women in rural areas. In most parts of Sub-Saharan Africa rapid growth of urban areas without adequate planning has caused pressure on water supply systems, resulting in water shortages for the entire region (14, 37). Whether HIV-positive women in Sub-Saharan Africa reside in rural or urban areas, they are faced with the challenge of access to potable water that may affect their ART adherence. A woman who experiences diarrhea as a side effect of ART but lacks water to drink, flush her toilet or wash herself, would likely have low adherence intention. Similarly, women without access to water for food preparation may be hesitant to take ART because they need to eat well in order to prevent medication side effects (26, 38, 39).

Although we examined ART adherence intention, our results are aligned with the Theory of Planned Behavior and are consistent with studies of socio-economic status and actual adherence behavior. Our results further inform findings from prior studies suggesting that women in a higher socio-economic status, as defined by education level or household income, were more likely to adhere to ART than women in a lower socio-economic status (21, 40–42). We found a weak relationship with income in bivariate analysis, but once residential location and other confounding variables such as water source were included in the model, income was no longer significant.

Our findings indicate that women with more HIV transmission knowledge were more likely to have high adherence intention compared to women with less HIV transmission knowledge, even after controlling for other factors. These findings support previous reports that people living with HIV are more likely to adhere to ART when they have a good knowledge of the cause of the disease and understand the effectiveness of ART (9, 43, 44). Generally, women in this study had good HIV transmission knowledge, which could be partly due to the pre- and post-test counseling conducted with them before enrolling in the treatment regimen. This finding is also aligned with previous reports that knowledge about HIV and AIDS has been increasing since 2003 in Sub-Saharan Africa, with 98% of women reportedly aware of the route of transmission and prevention of HIV (4, 45). Our results are also consistent with a recent study by Ramadhani et al. (46), who reported that patients made aware of their ART treatment guidelines and informed of the importance of their treatment were 10–20% more likely to adhere to their medications than patients who were not aware or informed.

### Limitations and Strengths

This study has some methodological limitations to consider when designing future studies. First, despite 100% agreement to participate in the study, our participants were selected through convenience sampling rather than random sampling. It is difficult to quantify how the non-random selection of the sample may affect the estimates, but it is likely that some bias was introduced. In addition, recruiting clients from clinics and hospitals might introduce some bias since most women who attend pre- and post-natal clinics are women who are most conscientious about their health and the health of their babies. Furthermore, all data for this study were self-reported. Self-reported measures of adherence behavior and adherence intention have been shown to overestimate the outcome (47, 48), although we encouraged participants to honestly answer all the questions.

Another limitation to consider in interpreting these results is that women were recruited if they had initiated ART at least 2 months prior to enrollment, however, we did not collect data on duration of ART, which could be associated with adherence intention. In addition, adherence intention was the outcome variable, rather than actual adherence behavior, which should be considered when interpreting the findings of this study. Although adherence intention was our outcome, our results are consistent with studies that look at socio-economic status and actual adherence (40, 49, 50). The Theory of Planned Behavior would also support the high consistency between intention and actual adherence behavior.

Despite the limitations, this novel study is the first known examination of the relationship between household water access and adherence intention among HIV-positive women receiving ART in both urban and rural districts of Zambia. Previous studies relied on other individual or structural level factors that affect adherence to ART, and were unable to capture the relationship between access to potable water and adherence intention. Therefore, this study serves as a baseline for comparisons with future studies. We believe that our study findings can be generalized to the larger population of women on ART in Zambia based on the similarities in representative national data regarding socio-demographic variables that capture both rural and urban environments. However, such generalization must be done with caution given the limitations of the study as sated above. In our bivariate analysis, source of water access and adherence intention were not significantly associated (PR 0.73, 95% CI: 0.52–1.02, *p* = 0.066). However, our sample had more access to piped water in our limited sample and studies with larger sample sizes are needed to show this association. In addition, future studies should more closely examine the relationship between household access to water and actual ART adherence behavior among pregnant and lactating women using more objective measures rather than self-report.

### Implications

Our study has important implications for public health practice and policy as it underscores the potential association between water access and ART adherence intention. Studying the environment and context of patients and populations have always been central to public health. Findings from this study reinforce the importance of assessing sources of accessible safe water when educating patients prior to initiating ART regimens rather than assuming that women with higher incomes or women living in urban areas have better access to potable water. The noted relationship between access to water and ART adherence intention may indicate that the efforts to improve access to water in low-resource countries may be more effective with specific population subgroups such as people living with HIV. For the implementation of the new ART regime “Treatment for All” to be successful, it is essential that social determinants of health for women and other persons living with HIV be taken into consideration.

There is paucity of studies that examine the association between water access and the health of women living with HIV in low-resource countries. Therefore, future research should replicate our findings in larger samples of men and women and expand on establishing how access to water influences ART adherence intention. These future studies should employ a mixed-methods approach, using population-level quantitative data in conjunction with individual-level qualitative data. There is also a need for multi-cultural comparisons that focus on countries or geographic regions with high proportions of vertical transmission of HIV to develop and test theory for a more holistic understanding of how water access influences adherence to ART in Sub-Saharan Africa (21).

## Data Availability Statement

The raw data supporting the conclusions of this article will be made available by the authors, without unduereservation.

## Ethics Statement

The protocol for this study was approved by Drexel University Institutional Review Board, with approval number 1706005436 and two Local Institutional Review Boards; the ERES Converge Institutional Review Board number 2017-Apr-011 and the Zambia National Health Research Authority number MH/101/23/10/1. Participants consented prior to enrollment using consent forms approved by Drexel University Institutional Review Board, ERES Converge Institutional Review Board, and Zambia National Health Research Authority. The patients/participants provided their written informed consent to participate in this study.

## Author Contributions

JN participated in the design of the research protocols, trained local staff on research protocols, analyzed all the research data, and participated in the drafting of the manuscript. JS-A participated in the design of the research protocol, drafting of the manuscript, and sought ethical approval at Drexel University. SM participated in research protocol, drafting of the manuscript, and mobilized funding for the project. TH participated in the analysis and review of the manuscript. RD-G, LJ, and FM participated in the design of the research protocol and drafting of the manuscript. EO participated in the design of the research protocol, sought ethical approval from Zambia, and supervised the research project locally in Zambia. All authors read and approved the final manuscript.

## Funding

This study was funded by Dornsife Global Development Scholar Program, Drexel University, as a dissertation grant to the JN.

## Conflict of Interest

EO was employed by World Vision Swaziland, Mbabane, Eswatini. The remaining authors declare that the research was conducted in the absence of any commercial or financial relationships that could be construed as a potential conflict of interest.

## Publisher's Note

All claims expressed in this article are solely those of the authors and do not necessarily represent those of their affiliated organizations, or those of the publisher, the editors and the reviewers. Any product that may be evaluated in this article, or claim that may be made by its manufacturer, is not guaranteed or endorsed by the publisher.
